# Gastrointestinal Motility and Improvement Efficacy of Shenhuang Plaster Application on Shenque: Identification, Evaluation, and Mechanism

**DOI:** 10.1155/2020/2383970

**Published:** 2020-07-11

**Authors:** Yanan Shi, Jingming Xu, Bin Ding, Guiping Chen, Lu Jin, Liang Ke, Xiao Xu, JingXia Wang, Qiuhua Sun, Xiaohong Xu

**Affiliations:** ^1^The College of Nursing, Zhejiang Chinese Medical University, Hangzhou 310053, China; ^2^The First Clinical Medical College, Zhejiang Chinese Medical University, Hangzhou 310053, China; ^3^College of Life Science, Zhejiang Chinese Medical University, Hangzhou 310053, China; ^4^The First Affiliated Hospital of Zhejiang Chinese Medical University, Hangzhou 310018, China; ^5^School of Second Clinical Medical, Zhejiang Chinese Medical University, Hangzhou 310053, China; ^6^The First Affiliated Hospital of Nanchang University, Nanchang 830052, China

## Abstract

Constipation, a gastrointestinal function disorder, is one of the side effects of paclitaxel (PTX) chemotherapy. Shenhuang plaster (SHP) application on the Shenque acupoint promotes gut motility in clinical settings. In this study, we elucidated the ingredients in SHP and evaluated its effects on PTX-induced constipation using a tumour-bearing mouse model. SHP was prepared using the traditional Chinese plaster preparation method. The ingredients were analysed using UPLC-MS/MS and identified via screening in a standard drug database. The gastrointestinal transit was evaluated by the movement of a fluorescein-labelled dextran in the gastrointestinal tract. A histological study of the mucosa was carried out after haematoxylin and eosin staining. mRNA expression was assessed using real-time RT-PCR, and the foetal microbiota composition was elucidated through 16 s rDNA sequencing and BLAST analysis. Our results indicate that the application of SHP attenuated weight gain inhibition by PTX; however, no inhibitory effect was observed on tumour growth. PTX-induced sluggish intestine, villus, and mucosal base layer damage were significantly improved following the application of SHP. Further, SHP enhanced the stimulation efficiency of PTX on TLR4 and its downstream cytokines, as well as on IL-1*β* in intestinal cells. SHP combined with PTX reshaped the microbiota, which showed beneficial effects on health. Hence, these results provide evidence that SHP alleviates PTX-induced constipation and intestinal morphological damage but augments the effects of PTX on the expression of cytokines in the TLR4 pathway and IL-1*β*. Therefore, we propose that SHP stimulates the host immune response to eradicate cancer cells.

## 1. Introduction

Constipation is a common clinical complication experienced during and after chemotherapy [1]. Breast cancer is the primary malignant tumour of the breasts. It has been identified as the most common malignant tumour in women [2]. In 2019, over 26,000 new cases were reported in the USA (2019, American Cancer Society, Inc., Surveillance Research) [3]. Chemotherapy remains a major treatment for breast cancer [4]. Some studies have indicated that anticancer medications, such as 5-fluorouracil-epidoxorubicin-cyclophosphamide, docetaxel, and paclitaxel increase the risk of constipation [1]. Prolonged constipation induces other complications, such as haemorrhoids, anal fissures, perianal abscesses, and rectal prolapse [5]. Measures which can alleviate constipation include the intake of fibre and fluids and an increase in physical activity. However, the positive effects of these measures are not immediate [6]. Chemotherapy is accompanied by other side effects, such as loss of appetite, vomiting, and weakened muscular strength [7] and various nonpharmacological approaches, such massage [8], acupuncture [9], and biofeedback [10] have shown positive effects.

External applications of herbal mixtures on Shenque (CV8), an acupoint, can improve bowel movement [11]. The use of external applications on Shenque (CV8) is a method that has been used for more than 1,000 years [12]. Traditional Chinese medical literature noted that CV8 is located in the “Ren” meridian and the joining point of “Ren”, “Du”, and “Sanjiao” meridians [13]. In Chinese medicine, these three meridians communicate to the 12 regular meridians and “Zang-fu” (internal organs) [14]. Therefore, Chinese doctors believe that the therapeutic treatment of the Shenque will stimulate the “Qi” (energy) circulating throughout the whole body [15].

We used Shenhuang plaster (SHP) application in CV8 therapy to improve constipation in breast cancer patients in the clinic with successful results [16]. Shenhuang plaster (SHP), consisting of Renshen (Ginseng Radix Et Rhizoma), raw Dahuang (Rhei Radix Et Rhizoma), Danshen (Salviae Miltiorrhizae Radix Et Rhizoma), Zhishi (Aurantii Fructus Immaturus), Houpo (Magnoliae Officinalis Cortex), Dingxiang (Caryophylli Flos), and Wuzhuyu (Evodiae Fructus), is an empirical formula, which has been used in clinics to ameliorate constipation for many years [17]. In this work, we used a paclitaxel-treated breast cancer mouse model to evaluate the medical activity and elucidate the pharmacological mechanism of SHP.

## 2. Materials and Methods

### 2.1. Chemical and Biochemical Materials

Paclitaxel (PTX, gyzz No. 20053001) was purchased from Yangzijiang Pharmaceutical (Taizhou, China); 4T1-luc cells were a gift from Dr. Gao (Zhejiang Chinese Medical University, China); isoflurane was purchased from Lunan Pharmaceutical Co., Ltd (Linyi, China). The 0.9% sodium chloride injection (120414-2) was purchased from Zhejiang Sapais Pharmaceutical (Jiaxing, China). Polyoxymethylene was purchased from the chemical Institute (Tianjin, China). Phosphate buffer solution (PBS) was purchased from Zhongshan Bio (Beijing, China). Anhydrous ethanol and haematoxylin and eosin were prepared by a chemical reagent company (Hangzhou, China). Ammonia was produced by CZ-chemical Plant (Hangzhou, China). Trizol, chloroform, isopropanol, DEPC water, and nuclease-free ddH2O were obtained from Ziker (Shenzhen, China). The genomic DNA, 5× HiScript and 4× gDNA, were purchased from Vazyme (Nanjing, China). The qPCR mix, 2×SYBR Green, was purchased from Fanbo Biochemicals (Beijing, China). Phusion Hot Start Flex 2× Master Mix (M0536L) was purchased from YiTao (Shanghai, China). NTP (4019), recombinant RNase inhibitor (2313A), reverse transcriptase M-MLV (2641A), and TB Green (RR420A) were obtained from TaKaRa (Dalian, China). DL2000 DNA Marker (3427A) was obtained from Takara Bio (Beijing, China). Qubit dsDNA HS Assay Kit (500 reactions) (Q32854) was purchased from Invitrogen (Carlsbad, CA, USA). AxyPrep PCR Cleanup Kit (35314KB1) was purchased from Axygen (Union City, CA, USA), Water DNA Kit (200) (D5525-02), Soil DNA Kit (200) (D5625-02), and Stool DNA Kit (200) (D4015-02) were purchased from OMEGA (Norcross, USA). Fluorescein-labelled dextran was purchased from Sigma-Aldrich (Sigma-Aldrich, St. Louis, MO, USA). The primers used in this study were synthesised by Sangon Biotech (Shanghai, China). Mass acetonitrile and methanol were purchased from Merck (Darmstadt, Germany); Chromatographic Grade 0.1% formic acid was purchased from Aladdin Information Technology Co., Ltd. (Hangzhou, China).

### 2.2. Equipment

The small animal anaesthesia machine used was obtained from Summit Industry (Illinois, USA). Stp120 dehydrator, ap280-2 embedding machine, and HM335e microtome were purchased from Thermo Fisher Microm (Walldorf, Germany). The Nikon Eclipse 80i microscope was purchased from Nikon (Tokyo, Japan). The Carl Zeiss imaging system was purchased from Carl Zeiss AG (Oberkochen, Germany). The microwave used was purchased from GalanZ (Guangdong, China). Our Allegra x-15r large capacity centrifuge was purchased from Beckman (CA, USA). The ultra-low temperature freezer (DW-HL388) was purchased from MeiLing. (HeFei, China). The NanoDrop One UV-Vis spectrophotometer and direct heat CO_2_ incubator (FormaTM 311) were purchased from Thermo Fisher Scientific (Waltham, MA, USA). The Super Clean Bench was obtained from SanXing Clean Technology (Suzhou, China). Our autoclave was purchased from Sanyo (Osaka, Japan). The Ag204 Electronic analytical balance was purchased from Mettler Toledo (Zurich, Switzerland). A LightCycler PCP was purchased from Roche (CA, USA). The 5417R centrifuge was purchased from Eppendorf (Hamburg, Germany). Our PCR instrument (A200 Gene Amplifier) was purchased from Longji Scientific Instrument Co. (Hangzhou, China). The vortex oscillator (WH-861 Vortex Shaker) was purchased from Hualida (Taicang, China). The NovaSeq sequencing platform was purchased from Illumina (USA) at LC Sciences Co., Ltd (Hangzhou, China). The SYNAPT G2-Si Q-TOF/MS quadrupole time-of-flight mass spectrometer, sample manager, and ACQUITY UPLC quaternary pump were purchased from Waters (Milford, MA, USA).

### 2.3. Preparation of Shenhuang Plaster

The ingredients in the SHP formula were 300 g (*Ginseng Radix Et Rhizoma*), 300 g raw Da Huang (*Rhei Radix Et Rhizoma*), 300 g Danshen (*Salviae Miltiorrhizae Radix Et Rhizoma*), 200 g Zhishi (*Aurantii Fructus Immaturus*), 250 g Houpo (*Magnoliae Officinalis Cortex*), 125 g Dingxiang (*Caryophylli Flos*), and 125 g Wuzhuyu (*Evodiae Fructus*). These Chinese herbal medicines were purchased from Chinese Herbal Pieces Co., Ltd. of Zhejiang Chinese Medical University and in compliance with 2015 Chinese Pharmacopoeia standards. The production steps were as follows: (1) ginsenosides were extracted from Renshen; (2) total anthraquinone was extracted from raw Da Huang; (3) 300 g Danshen, 200 g Zhishi, 250 g Houpo, 125 g Wuzhuyu, and 75 g Dingxiang were mixed and refluxed twice with eight times the amount of 80% ethanol, for 1 h each. Ethanol was recovered by freeze-drying until all the alcohol had disappeared from the solvent; (4) 50 g Dingxiang was crushed into coarse particles, added to 10 times the amount of water, soaked for 12 h, and extracted by steam distillation for 8 h. Then, aromatic water was collected. The powders of steps (1), (2), and (3) were mixed for uniformity and the extracted aromatic water from step (4) was used as a penetrant. After mixing, the SHP is prepared and stored at 4°C. The extraction of the ingredients and the preparation of the SHP were completed at the Traditional Chinese Medicine Preparation Room of Zhejiang Chinese Medical University.

### 2.4. UPLC-MS/MS Analysis

SHP was dissolved with methanol and centrifuged to obtain the supernatant. One microlitre of the supernatant was loaded into an ACQUITY UPLC BEH C18 column (100 × 2.1 mm, 1.6 *μ*m) by an ultra-high-performance liquid chromatography system. The chromatographic conditions included a Waters Acquity UPLC BEH C18 column (100 mm × 2.1 mm, 1.6 *μ*m) with a column temperature of 30°C, the mobile phase: 0.1% formic acid aqueous solution (A) and 0.1% acetonitrile solution (B). The gradient elution details are as follows: 0 to 2 min, 10% B; 2 to 26 min, 10% to 90% B; 26 to 28 min, 90% B; 28 to 28.1 min, 90% to 10% B; 28.1 to 30 min, 10% B. The injected sample volume was 1 *μ*L. The temperature of the auto-sampler was maintained at 10°C, and the flow rate was 0.3 mL/min. MS data was obtained using a SYNAPT G2-Si Q-TOF/MS spectrometer system in positive and negative ion mode by an electrospray ionisation mass spectrometer (ESI-MS/MS). The MS conditions were SYNAPT G2-Si Q-TOF/MS spectrometer, in MSE continuum mode, with a scan range of *m*/*z* 50 to 1500. In ESI+ and ESI− modes, the capillary voltage and the taper voltage were 3.0 kV and 40 V, and the taper hole flow rate was 50 L/h at 120°C. When the temperature was 400°C, the dissolvent flow rate was 800 L/h, and the measurement of mass was based on 1 ng/*μ*L leucine-enkephalin (ESI+: *m*/*z* 556.2771, ESI−: *m*/*z* 554.261 5) solution used as the calibration standard solution (Lock Spray TM).

### 2.5. 4T1-Luc Cells Suspension Preparation

The 4T1-Luc cells were grown in RPMI-1640 medium, which contained 10% foetal bovine serum, 50 IU/mL penicillin, and 50 *μ*g/mL streptomycin at 37°C in a 5% CO_2_, 100% humidity incubator. The medium was refreshed every two days, until a sufficient number of cells were obtained for the experiment. The cells were collected and washed three times with PBS. Finally, the cells were resuspended in PBS buffer (5 × 10^6^ cells per 1 mL PBS). This cell suspension was used for subcutaneous injection.

### 2.6. Animal Treatment

BALB/c female mice (8 weeks old, 18–20 g) were purchased from the Shanghai Laboratory Animal Centre (Shanghai, China). The mice were fed under specific pathogen-free conditions (SPF) at the animal experimental centre of Zhejiang Chinese Medical University, with a strict light/dark cycle (12 hours of light), at 20°C and with unrestricted access to food and water. All the animal experiments were approved by the committee of and performed at Zhejiang Chinese Medical University Animal Research Centre (Accepted Nr. 11039). All the mice were anesthetised by inhalation of 4% isoflurane. The mice were subcutaneously injected with 100 *μ*L of 4T1-Luc cell (5 × 10^5^) PBS suspension into the fourth right breast. At 10 days post tumour cell implantation, the diameters of the primary tumours were measured. The mice with the same size of the tumour were randomly separated into four groups (at least six mice in each group): (1) the control group (Ctrl, on CV8 administration of 100 *μ*L saline/mouse/day, and intraperitoneal injection of 0.1 mL saline/mouse/every other day), (2) the SHP group (on CV8 administration of 100 *μ*L 500 mg SHP/mL/mouse/day, and intraperitoneal injection of 0.2 mL saline/mouse/every other day), (3) the PTX treated group (on CV8 administration of 100 *μ*L saline/mouse/day, and intraperitoneal injection of 18 mg/kg body weight in 0.2 mL/mouse/every other day), and (4) the PTX+SHP group (on CV8 administration of 100 *μ*L 500 mg SHP/mL/mouse/day, and intraperitoneal injection of 18 mg/kg body weight in 0.2 mL/mouse/every other day). Normal saline was used in this process to eliminate the differences in the treatment. At the beginning of the third week, the day after the last injection, the stool of each mouse was collected separately, in a biosafety cabinet. Then the gastrointestinal transit was evaluated. At the end, the colon and ileum were collected and separated into two parts; one fixed with 4% neutral buffered formalin, the other frozen in liquid nitrogen.

### 2.7. Measurement of Gastrointestinal Transit

The mice were deprived of food for 24 h, and each mouse was given 200 *μ*L of 6.25 mg/mL fluorescein-labelled dextran (70 kDa) by gavage administration. After 30 min, the mice were put in a CO_2_ chamber to be sacrificed. The excision of the gastrointestinal tract was divided into 15 segments including the stomach (S1), small intestine from the stomach to cecum (from S2 to S11), cecum (S12), and colon (S13, S14, and S15). The fluorescence intensity in each segment was identified at 494 nm/521 nm with a fluorescence spectrophotometer. The geometric centre (GC) of the fluorescence distribution, which was indicated as the centre of gravity for the distribution of fluorescein, was calculated using the following formula: GC = *Σ* (%of total fluorescent signal per segment∗segment number)/100.

### 2.8. Histologic Examination

Histochemical examination was performed on whole-mount muscularis preparations of the ileum and colon after haematoxylin and eosin (H&E) staining. The formalin-fixed organ parts (5 mm) were dehydrated by consecutive application of increasing concentrations of xylene (10–100%) in alcohol. The dehydrated organ parts were separately embedded in paraffin and cut into 4 *μ*m thin slices. The slices were dried at 65°C in an oven for one hour. The paraffin was washed with xylene, a concentration gradient decrease of alcohol solution, and sterile deionised water. Finally, the slices were stained in H&E solution for 5 min, followed by dehydration with alcohol and xylene treatment. These stained slices were imaged with the NanoZoomer Digital Slice Scanner (NDP) and analysed with the specific software supplied by NDP.

### 2.9. Reverse Transcriptase and Real-Time Quantitative PCR

The total RNA was extracted with Trizol/chloroform method, according to the Trizol Reagent manual supplied by Invitrogen. The quality and concentration of isolated mRNA was determined by absorbance ratio at 260 nm and 280 nm, measured with a Nanodrop 2000. One microgram of total RNA was reverse transcribed; cDNA was used as template for real-time PCR. The PCR conditions were as follows: template denaturation at 95°C for 5 min and 45 amplification cycles of 95°C for 10 s, 55°C for 30 s, and 72°C for 10 s. The melting curve was monitored as the temperature increased from 65°C to 95°C. The target proteins and the specific primer pairs are listed in Table 1.

### 2.10. 16S rDNA Gene Sequencing and Data Analysis

The genomic DNA was extracted from 200 mg of stool using the phenol-chloroform method. The quality and concentration of the extracted DNA was determined by the absorbance ratio at 260 nm and 280 nm and electrophoresis. The Phusion Hot Start Flex 2× Master Mix was employed for PCR amplification of the V3-V4 region of the 16S rDNA gene with a universal primer pair (341F 5′-CCTACGGGNGGCWGCAG-3′, 805R 5′- GACTACHVGGGTATCTAATCC-3′). The Qbuit and Agilent 2100 Analyser were used to quantify the library and evaluate the library quality. An insertion sequence (275–450 bp) was selected and sequenced via the NovaSeq sequencing platform. According to the specific barcode of the sample, the paired-end data were overlapped using the FLASH software (Version 1.2.11). Fqtrim (v0.94) was employed to remove the low-quality sequence and host pollution sequence and to obtain the clean data. The data were filtered with Vsearch (v2.3.4). Further, the specific features and sequences were obtained after DADA2 (Divisive Amplicon Denoising Algorithm) and ASV analysis. The diversity was calculated by normalising to the same random sequence. The characteristic abundance of each feature was normalised using its relative abundance with the SILVA (release 132) classifier. The feature was clustered into operational taxonomic units (OTUs) at 97% similarity by BLAST.

After Soap de novo (v2.04) was used to assemble and analyse the selected clean data, MetaGeneMark was used to predict genes and construct nonredundant gene sets; MyTaxa and related databases were used to obtain species annotation information of each gene, and combined with the gene abundance table, species abundance tables of different classification levels were obtained. Based on the relative abundance of OTUs at each classification, R software was used to generate the heat map.

### 2.11. Statistical Analysis

In this study, every group had at least four mice, and each value is represented by mean ± SD. The differences between the groups were analysed using one-way ANOVA model by SPSS software (version 19.0, SPSS). A *p* value of less than 0.05 was considered statistically significant.

## 3. Results

### 3.1. UPLC-MS/MS Analysis of Chemical Constituents of SHP

**Figure 1:**
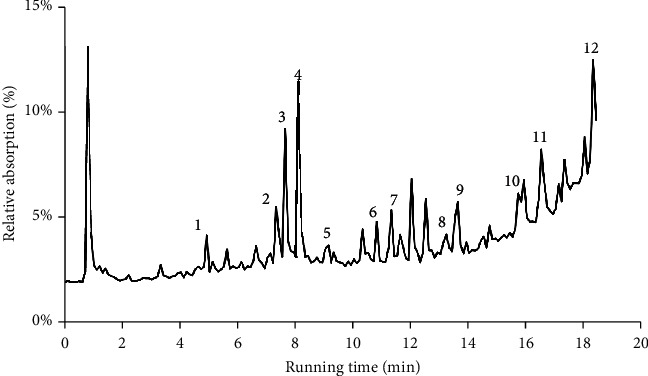
UPLC-MS/MS analysis of Shenhuang plaster (SHP) in positive ionisation mode. The retention time of identified main appeared peaks was between 0 and 18 min in total ion current (TIC) chromatogram. The specific ingredients of SHP are marked from 1 to 12. The *m*/*e* value (Measured and Theoretical value) and retention time of the identified peaks are listed in Table 2.

SHP, a seven-herb mixture extract containing hundreds of components, was prepared using traditional methods. In this study, the ingredients in SHP were validated with UPLC-MS/MS, analysed in both positive and negative ionisation modes, and combined with in-house database screening. Twelve specific ingredients were found, which are marked in Figure 1 and noted in Table 2. Most of these components were found in both modes. However, salvianic acid A (retention time = 3.31 min) was found only in the negative mode. The indicated herbs are available in a drug standard database https://www.drugfuture.com/standard/ (In Chinese).

### 3.2. SHP Alleviated PTX Induced Bodyweight Loss

The changes in the bodyweight of the tumour-bearing mice were monitored in this research. The initial bodyweight of each mouse was measured 14 days after tumour-cell inoculation. From the 15th day, the mice in the Ctrl, SHP, PTX, and PTX+SHP groups were treated as described in the methods section. After the final treatment, the bodyweight of each mouse was measured again. The average amount of weight gain in each group was calculated and is listed in Table 3 as a *D*-value. The mice in three groups (Ctrl, SHP, and PTX+SHP) gained bodyweight, while no obvious changes were observed in the bodyweight of those in the PTX group. Significant differences between PTX, PTX+SHP, and Ctrl were found (^∗∗∗^*p* < 0.001, ^∗∗^*p* < 0.01). Additionally, in comparison with the PTX group, the weight gain in PTX+SHP group was significant (^△^*p* < 0.05).

### 3.3. SHP Enhanced the Inhibitory Effect of PTX on Tumour Growth

**Figure 2:**
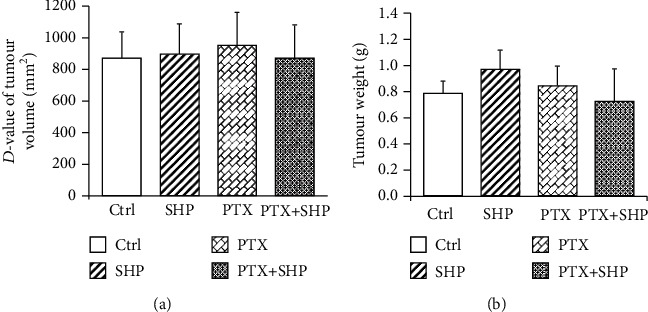
Tumour growth. (a) Changes in tumour volume (*D*-value) (b) in each group. The differences between groups were comparatively analysed using one-way analysis of variance (ANOVA) and Bonferroni multiple comparison test. No statistical differences were observed.

Tumour growth was represented as changes in tumour volume, as well as tumour weight. The average tumour weight in the Ctrl, SHP, PTX, and PTX+SHP groups was 0.79 ± 0.09, 0.97 ± 0.14, 0.85 ± 0.13, and 0.72 ± 0.25 g, respectively. The average tumour volume change in each group was measured and calculated as *D*-value. The *D*-values of Ctrl, SHP, PTX, and PTX+SHP groups were 864.35 ± 170.33, 898.08 ± 188.13, 951.95 ± 207.21, and 973.64 ± 250.68 mm^2^, respectively. Further, there were no statistical differences in the tumour weight and changes of tumour volume between any two groups. Interestingly, the tumour in PTX+SHP was the lightest and the smallest (Figure 2).

### 3.4. SHP Improved PTX-Induced Gastrointestinal Motility Disorder

**Figure 3:**
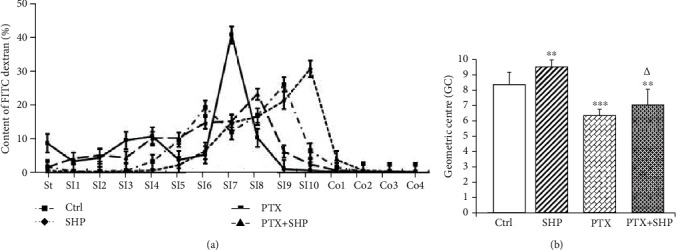
Intestinal motility evaluation. (a). Transit curves: the distribution of fluorescein-labelled dextran in the gastrointestinal tract (St: stomach; SI: small intestinal segments from 1 to 10; Co: colon segments from 1 to 4) of mice in each group. (b) Derived geometric centre (GC) of mice of each group. GC refers to that part of the gastrointestinal tract, which shows the highest fluorescence signal. The significant difference between each group and Ctrl or PTX is denoted as ^∗^ (^∗∗^*p* < 0.01; ^∗∗∗^*p* < 0.001) or *^Δ^* (*^Δ^p* < 0.05), respectively.

The gastrointestinal motility was comparatively evaluated based on fluorescence residual rates in various parts of the alimentary canal. The fluorescently labelled dextran (70 kDa) was aborally transported in the Ctrl groups, with a maximal signal detected in the SI9 (at ileum terminal). Gastrointestinal motility in the SHP group, which showed a maximum signal level in SI10, was faster than that in the Ctrl group. In contrast, dextran signal levels in PTX and PTX+SHP groups were accumulated in SI7 and SI8 (jejunum), respectively (Figure 3(a)). The calculated geometric centre (GC) of each group is shown in Figure 3(b). The GC of PTX, PTX+SHP, Ctrl, and SHP were 6.31 ± 0.31, 7.04 ± 0.12, 8.34 ± 0.81, and 9.52 ± 0.08, respectively. This indicated that the relation between the groups in terms of the speed of gastrointestinal motilities was PTX < PTX + SHP < Ctrl < SHP. As shown in Figure 3(b), the GC of PTX+SHP was significantly different from that of PTX (*p* < 0.05).

### 3.5. SHP Protected the Integrity of Intestinal Morphology

**Figure 4:**
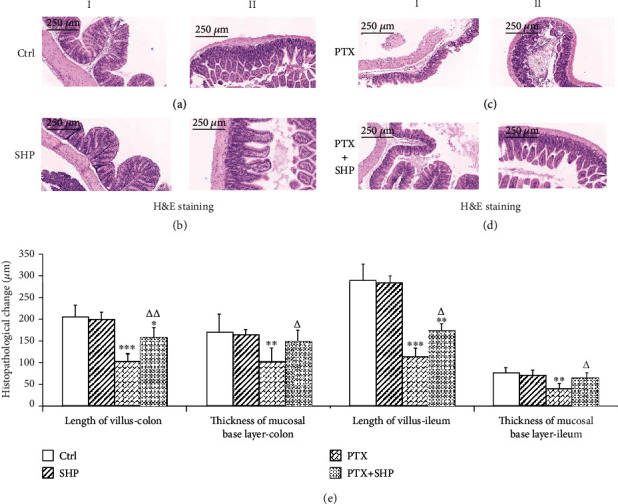
H&E staining and histological study. Histological study of (I) colon and (II) ileum from (a) Ctrl, (b) SHP, (c) PTX, and (d) PTX+SHP groups. (e) Average length of intestinal villus and average thickness of the mucosal base layer in the colon and ileum from mice of each group, which were measured with NDP view software. The difference between each group and Ctrl is marked with ^∗^ (^∗^*p* <0.05; ^∗∗^*p* < 0.01; ^∗∗∗^*p* < 0.001). The difference between PTX+SHP and PTX groups is marked with *^Δ^* (*^Δ^p* < 0.05; *^ΔΔ^p* < 0.01).

The length of villi and thickness of the muscular layer, two major intestinal morphology parameters, are indicative of gut health. Therefore, in our research, the colon (Figures 4(a)–4(d), part I) and ileum (Figures 4(a)–4(d), part II) in different groups were H&E stained and observed with a microscope. SHP treatment did not affect the length of the villi or the thickness of the muscular layer. In comparison, disrupted villi and muscular layer in PTX were clearly observed under a microscope. As shown in Figure 4(e), the villi length from both colon and ileum was significantly different between each group and Ctrl (^∗^*p* < 0.05; ^∗∗^*p* < 0.01; ^∗∗∗^*p* < 0.001). In addition, the ileal and colic villi lengths in PTX+SHP were greater than in PTX (*^Δ^p* < 0.05). The thickness of the ileal and colic muscular layers were statistically different between PTX and Ctrl groups (^∗∗^*p* < 0.01) as well as between PTX and PTX+SHP groups. However, no statistical difference was observed between that of PTX+SHP and Ctrl groups.

### 3.6. SHP and PTX Regulated the Transcription of some Intestinal Cytokines

**Figure 5:**
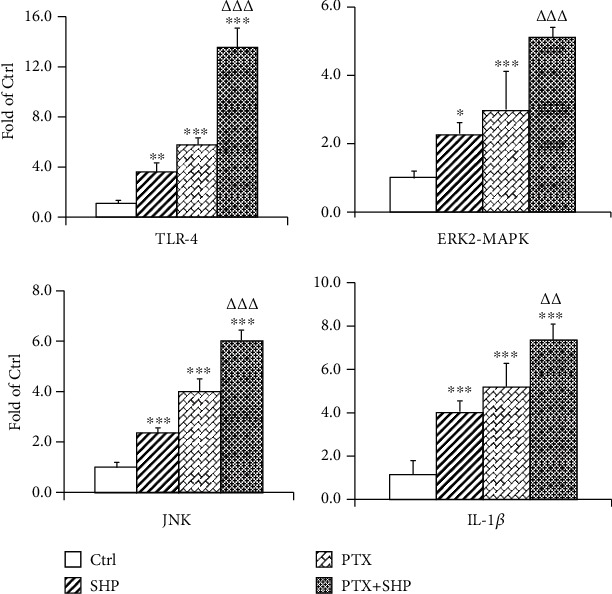
Relative expression of some inflammatory cytokines and signal molecules in the ileum of mice from different groups. The gene expression of TLR-4, ERK2-MAPK, JNK, and IL-1*β* in each group was determined by real-time PCR with 18 s rRNA as reference gene. Data are presented as fold change compared to that of the Ctrl group. The statistic differences between the group and Ctrl or PTX are marked with ^∗^ (^∗^*p* < 0.05; ^∗∗^*p* < 0.01; ^∗∗∗^*p* < 0.001) or *^Δ^* (*^Δ^p* < 0.05; *^ΔΔ^p* < 0.01; *^ΔΔΔ^p* < 0.001), respectively.

The transcription of some proinflammatory cytokines in intestinal tissue from different groups was investigated using real-time PCR. The results were statistically analysed and are presented in Figure 5. SHP and PTX treatments improved the expression of JNK, ERK2-MAPK, IL-1*β*, and TLR-4 in intestinal cells. This improvement in the capability of PTX was stronger than that of SHP alone. Further, these improvement effects were superimposed on the PTX+SHP group.

### 3.7. SHP Influenced Foetal Microbiota

**Figure 6:**
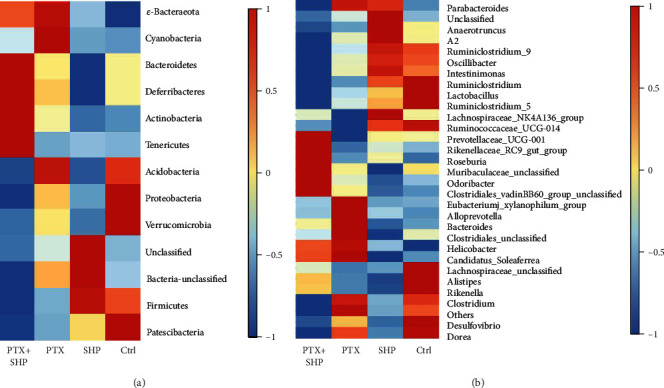
Species abundances in four different groups according to 16 s rRNA gene sequencing. Each row of (a) represents a bacterial phylum and (b) represents the colony abundance of each genus. Different colours represent the degree of aggregation of abundance.

Finally, the effect of SHP on foetal microbiota was determined by sequencing the V3-V4 region of 16 s rDNA. Over 1201 thousand clean data were obtained by PCR. There are 1851 unique OTUs in the Ctrl, 2086 in SHP, 1913 in PTX, and 2500 in PTX+SHP. 879 OTUs in the PTX group were shared by PTX+SHP group, which are indicated using a Venn diagram. The richness and diversity of the microbiota community in the groups were evaluated by *α*-diversity analysis. No specific differences were found between groups (*p* < 0.06). The diversity of the microbiota community between different groups were evaluated with the *β*-diversity analysis. The bacteria in phylum Patescibacteria, Proteobacteria, and Verruomicrobia were enriched in the Ctrl group, but infertile in SHP and PTX+SHP groups. Firmicutes, a BMI gain phylum, and many unclassified bacteria were enriched in SHP but infertile in PTX+SHP. Actinobacteria, Cyanobacteria, and *ε*-Bacteraeota were enriched by PTX. The bacteria in phylum Bacteroidetes, Deferribacteres, Actinobacteria, and Tenericutes, which were infertile in the other three groups, were enriched in the PTX+SHP group. The bacterial density at both the phylum level as well as at the genus level in different groups was variform (Figures 6(a)–6(b)). At the genus level, Prevotellaceae UCG-001, Rikenellaceae_RC9_gut_group, *Roseburia*, Muribaculaceae_unclassified, *Odoribacter*, and *Clostridiales* unclassified were enriched in PTX+SHP. *Ruminiclostridium*, Oscillibacter, *Intestinimonas*, *Ruminiclostridium*, *Lactobacillus*, *Ruminiclostridium*, Lachnospiraceae_NK4A136_group, and Ruminococcaceae_UCG-014 were infertile in PTX+SHP but enriched in SHP and Ctrl groups. However, the bacterial density in the PTX group was still higher than that in PTX+SHP. The enriched Eubacteriumj_xylanophilum_group, Alloprevotella, Bacteroides, Clostridiales_unclassified, Helicobacter, and Candidatus_Soleaferrea in PTX were infertile in both SHP and PTX+SHP group. The genus *Clostridium*, *Desulfovibrio*, and *Dorea*, enriched in PTX, were more infertile in PTX+SHP than that in SHP.

## 4. Discussion

Constipation is a common side effect of paclitaxel, a drug, prescribed during breast cancer treatment [18]. Patients in China are commonly treated using complementary and integrative therapies, especially traditional Chinese medicine (TCM). The application of SHP on Shenque (CV8) showed good clinical effects by relieving constipation resulting from postoperative ileus (POI) [19] or hepatic diseases [20]. In this study, we first elucidated the components in SHP by UPLC-MS/MS. The pharmaceutical effect of SHP application on Shenque (CV8) was evaluated with a tumour-bearing mouse model. The application of SHP on Shenque did not induce diarrhoea or weight loss. On the contrary, SHP attenuated the paclitaxel treatment-induced tumour weight changes. Tumour growth differed among groups. However, no significant differences were found between any two groups (Figures 2(a)–2(b)). The beneficial effects of SHP (powder) application on gastrointestinal motility were evaluated with different postoperative ileus (POI) animal models in our lab [16, 21–23]. In clinical settings, SHP has been used to ameliorate constipation in breast cancer patients for many years. This is the first time this process has been studied using tumour-bearing mice. Similar to other models, the effects of SHP were significant (Figures 3(a)–3(b)). The morphological changes in the intestinal villi and the mucus layer, as well as the thickness of the muscular layer in PTX, were obviously observed, in comparison with those in Ctrl and SHP groups. In addition, SHP protected the ileum and colon from the toxic effects of PTX, which led to morphological changes in the intestine (Figures 4(a)–4(e)). Recent studies suggested that the PTX may induce cell inflammation [24, 25]. Therefore, we elucidated the regulatory activity of SHP (and combined with PTX) on the transcription of some proinflammation molecular and signalling factors. TLR-4, ERK2-MAPK, JNK, and IL-1*β* in intestinal cells were upregulated (Figures 5(a)–5(d)). Flora is a key regulator in constipation, which can be altered with different treatments (Figures 6(a)–6(b)).

Generally, prescribed formulas in TCM involve a complex herbal system, which contains hundreds or even thousands of different chemical ingredients. The formula of SHP was developed from Dachengqi decoction (major order the qi decoction) by a nationally recognised Chinese medicine doctor, named Xu, Zhiying [26]. Dahuang (*Rhei Radix Et Rhizoma*), an herb in the SHP, has been used frequently for hundreds of years in China to treat constipation [27]. Aloe-emodin and emodin in Dahuang are herbal components well known for modulating the contractility of intestinal smooth muscle [28, 29]. In our previous research, the absorption and pharmacokinetics of SHP application on Shenque (CV8) were evaluated. Emodin and magnolol (from Houpo, *Magnoliae Officinalis Cortex*) were found in the plasma of SHP-applied rabbits [26]. Magnolol from Houpo (*Magnoliae Officinalis Cortex*) can significantly promote gastrointestinal movement [30]. Otherwise, it was demonstrated by previous studies that ginsenoside Rd and Rg1 from Renshen (*Ginseng Radix Et Rhizoma*), as well as tanshinone IIA in Danshen (*Salviae Miltiorrhizae Radix Et Rhizoma*), positively affect the intestinal flora and protect the mucus layer [31] of small intestines. In this study, the components with beneficial effects on gastrointestinal motility were identified with UPLC-MS/MS. We proposed these ingredients were the pharmaceutic basis of SHP for clinic effects.

According to a report from the American Society of Clinical Oncology, about 40% of diagnosed cancer patients undergo unexplained weight loss [32]. However, in this study, the tumour-bearing mice actually gained weight. On the contrary, PTX treatment reduced weight gain significantly. This indicates the toxicity of PTX [33, 34]. Obviously, the application of SHP prevented this tendency (Table 3). This is an advantageous attribute of SHP treatment. Breast cancer diagnosis and chemotherapy treatment may last months to years. Patients are required to ingest multiple drugs, not only for symptom management but also for side effect management. In China, acupuncture and moxibustion, massage, cupping, as well as medical powder or plaster, are popularly used alternative therapies [35]. One of the benefits of these therapies is that they help to avoid some unknown interactions of pharmaceutical drugs. In this study, we found that SHP applied on CV8 enhanced the chemotherapeutic efficiency of paclitaxel, which had more effect on the tumour size and the weight of PTX+SHP, compared with the other three groups.

In this research, morphological damage of the gastrointestinal villi and mucus layer was observed in the PTX group but not in the other three groups. PTX as a chemotherapy drug for breast cancer treatment induces tumour cell apoptosis by stabilising microtubules [36], blocking cell division [37], and inducing cells death (at high concentrations) [38]. It was demonstrated that PTX induces apoptosis through the TLR-4-mediated [39, 40] ERK2-MAPK signalling pathway [41] and stimulates the expression of some inflammatory molecules, such as IL-1*β* [33]. Therefore, in our research, we evaluated the regulatory capability of SHP and PTX+SHP on TLR-4 and the downstream molecules. The SHP group represents an activity similar to that of PTX, which stimulated the transcription of TLR-4, ERK2-MAPK, JNK, and IL-1*β*. Further, in the PTX+SHP group, the regulatory activities of PTX and SHP seem to be mutually reinforced (Figure 5). In fact, TLR-4-mediated MAPK pathway is mainly responsible for innate immune responses [42]. It is one of the characteristic molecules of host cell defences against pathogens, immune cell regulation, survival, and proliferation [43–46]. Although inflammatory cytokines are implicated in tumorigenesis and tumour progression in breast cancer [47–49], they are also being investigated for their ability to control cancer progression. Histological observations indicated no significant damage or inflammation of the ileum or colon. We proposed that the PTX combined with SHP application evoke an immune reaction against the tumour, but it does not lead to the apoptosis of intestinal cells.

Microflora is at the very core of the chemotherapeutic response occurring outside of the intestine [50, 51]. LPS of Flora bacteria can affect the transcription of TLR-4/MAPK and IL-1*β*. TLR-4 and IL-1*β* are overexpressed in the intestinal epithelium of patients with Crohn's disease (diarrhoea) [52, 53]. Our research demonstrated that SHP application on CV8 altered the microbiome community. The species identified in the faecal samples of PTX+SHP-treated tumour-bearing mice were characterised by *α*-diversity and *β*-diversity analysis. Certain bacteria which are beneficial in chemotherapy have been identified. For instance, Bacteroides [54], Deferribacteres [55], Tenericutes [56], and Actinobacteria [57] were indicated to be of beneficial for chemotherapy patients. At the genus level, Prevotellaceae_UCG-001 [58] and Rikenellaceae_RC9_gut_group [59] are involved in the degradation of carbohydrates and proteins to produce various organic acids. In addition, *Roseburia* [60] and *Odoribacter* [61], species from *Bacteroides*, are butyrate-producing gut bacteria [62]. The abundance of Clostridiales_vadinBB60_ group uncultured bacteria are closely related to faecal short-chain fatty acid production [63]. These five genera were specifically enriched in the PTX+SHP group (Figure 6). Short-chain fatty acids, especially butyrate, affect gut-motility [64], health [62], and the immune system [65], and also benefit the microflora metabolites. Results of the microbiota analysis supported our hypothesis that the microbiota of the PTX+SHP group did not induce gut-inflammation but protected intestinal morphology and function. However, the reason for the effects of PTX+SHP on cytokine transcription in the TLR-4 pathway and IL-1*β* is unknown. Hence, we, again, hypothesise that PTX+SHP combination promotes the immune response of tumour-bearing mouse. This hypothesis will be elucidated in our future research.

## 5. Conclusion

In conclusion, data from the present research indicate that SHP is a gut motility-promoting candidate in CV8 application. SHP application protected intestinal villi and the mucosa layer from cell toxicity originating from PXT. Moreover, these benefits may rely on the PTX+SHP-specific gut microbiota. Finally, the combination of PTX and SHP stimulated the immune response, suppressing tumour development.

## Figures and Tables

**Table 1:** Real-time PCR primer sequence.

Target cytokines	Forward (5′ to 3′)	Reverse (5′ to 3′)
TLR-4	TGGATACGTTTCCTTATAAG	GAAATGGAGGCACCCCTTC
ERK2-MAPK	TCAGATGAATTTTCGTTGGCAGA	AGCTTTTGTATTGGTCACAGCA
JNK	AGCCGTCTCCTTTAGCACAG	TGTATCCGAGGCCAAAGTCG
IL-1*β*	GAAATGCCACCTTTTGACAGTG	TGGATGCTCTCATCAGGACAG

**Table 2:** Peak assignments of specific ingredients in SHP via UPLC-MS/MS.

PK. No.	Rt (min)	Elemental composition	*m*/*e*		Adduct	Identification	Indicate herb
			Measured value	Theoretical value			
1	4.90	C19H20O3	314.1748	296.1412	NH4	Tanshinone II B	Danshen (*Salviae Miltiorrhizae Radix Et Rhizoma*)
2	7.26	C15H10O5	271.0597	270.0528	H	Emodin	Dahuang (*Rhei Radix Et Rhizoma*)
3	7.63	C28H34O15	611.2066	611.1976	H	Hesperiden	Zhishi (*Aurantii Fructus Immaturus*)
4	8.07	C42H72O14	823.4794	800.4922	Na, H, K	Ginsenoside Rg1	Renshen (*Ginseng Radix Et Rhizoma*)
5	9.06	C15H10O5	271.0600	270.0582	H	Aloe-Emodin	Dahuang (*Rhei Radix Et Rhizoma*)
6	10.78	C19H20O3	297.1481	296.1412	H	Isocryptotanshinone	Danshen (*Salviae Miltiorrhizae Radix Et Rhizoma*)
7	11.33	C48H82O18	969.5390	946.5501	Na, H, NH4	Ginsenoside Rd	Renshen(*Ginseng Radix Et Rhizoma*)
8	13.20	C19H17N3O	304.1439	303.1372	Na, H	Evodiamine	Wuzhuyu (*Euodiae Fructus*)
9	13.60	C11H14O4	211.0964	210.0892	H	2,4,6-Methoxyacetophenone	Dingxiang (*Caryophylli Flos*)
10	15.86	C18H18O2	267.1376	266.1307	H	Magnolol	Houpo (*Magnoliae Officinalis Cortex*)
11	16.47	C15H10O4	255.0651	254.0579	H	Chrysophanol	Dahuang (*Rhei Radix Et Rhizoma*)
12	18.29	C9H13NO	174.0888	151.0997	Na	4-(2-(Methylamino)ethyl)phenol	Zhishi (*Aurantii Fructus Immaturus*)

**Table 3:** The bodyweight gain of mice in each group (mean ± SD).

Group	Initial bodyweight	Final bodyweight	*D*-value
Ctrl	21.10 ± 0.36	23.24 ± 1.15	2.38 ± 0.77
SHP	21.23 ± 0.62	22.83 ± 0.39	1.60 ± 0.49
PTX	21.23 ± 0.93	21.45 ± 0.88	0.23 ± 0.10^∗∗∗^
PTX + SHP	20.80 ± 1.51	21.83 ± 1.82	1.03 ± 0.50^∗∗^^△^

^∗^Statistical difference between the indicated group and Ctrl group (^∗∗^*p* < 0.01; ^∗∗∗^*p* < 0.001). ^△^ represents the difference between PTX and PTX+SHP group (^△^, *p* < 0.05).

## Data Availability

We have submitted our recent research titled “Gastrointestinal motility and improvement efficacy of Shenhuang Plaster application on Shenque: identification, evaluation, and efficacy of Shenhuang Plaster application on Shenque: identification, evaluation, and available and accessible online. We have annotated the entire data building process an empirical techniques presented in the paper. All data used to support the findings of this study are included within the article and these data also can be accessible on website https://www.force11.org/article/gastrointestinal-motility-and-improvement-efficacyshenhuang-plaster-application-shenque. We are willing to authorize the editor from “Journa of Immunology Research” update the data on website. If the “raw” data are needed, please contact with the corresponding author Prof. Xu.
